# Graduate health professions education programs as they choose to represent themselves: A website review

**DOI:** 10.12688/mep.19498.1

**Published:** 2023-03-03

**Authors:** Janse Schermerhorn, Shelby Wilcox, Steven Durning, Joseph Costello, Candace Norton, Holly Meyer

**Affiliations:** 1 Medical Education, Uniformed Services University of the Health Sciences, Bethesda, Maryland, 20814, USA; 2 Orthopedics, Walter Reed National Military Medical Center, Bethesda, Maryland, 20814, USA; 3 Graduate Medical Education, Naval Medical Center Portsmouth, Portsmouth, VAA, 23708, USA

**Keywords:** Website, HPE, Program values.

## Abstract

**Introduction:** In an age of increasingly face-to-face, blended, and online Health Professions Education, students have more choices of institutions at which to study their degree. For an applicant, oftentimes, the first step is to learn more about a program through its website. Websites allow programs to convey their unique voice and to share their mission and values with others such as applicants, researchers, and academics. Additionally, as the number of health professions education (HPE) programs rapidly grows, websites can share the priorities of these programs.

**Methods:** In this study, we conducted a website review of 158 HPE websites to explore their geographical distributions, missions, educational concentrations, and various programmatic components.

**Results:** We compiled this information and synthesized pertinent aspects, such as program similarities and differences, or highlighted the omission of critical data.

**Conclusions:** Given that websites are often the first point of contact for prospective applicants, curious collaborators, and potential faculty, the digital image of HPE programs matters. We believe our findings demonstrate opportunities for growth within institutions and assist the field in identifying the priorities of HPE programs. As programs begin to shape their websites with more intentionality, they can reflect their relative divergence/convergence compared to other programs as they see fit and, therefore, attract individuals to best match this identity. Periodic reviews of the breadth of programs, such as those undergone here, are necessary to capture diversifying goals, and serves to help advance the field of HPE as a whole.

## Introduction

The age-old adage, ‘see one, do one, teach one’ has expired. In its wake, health professions education (HPE) has emerged. HPE programs equip health care providers with the academic prowess, interpersonal skills, and leadership to advance the field, because students taught by skilled clinical educators often perform better in their fields ^1^. Furthermore, learning the educational and leadership skills necessary to teach requires practice and a significant time commitment ^2, 3^. Medical personnel are often constrained in their capacity to teach effectively due to busy clinical schedules ^4^. HPE programs have come to meet this need and to serve as a reference standard to ensure health professionals are equipped with skills in education, leadership and research despite different locations, styles, and contents ^5^.

In an age of constantly improving technology, and in the context of the severe acute respiratory coronavirus 2 (SARS-CoV-2) pandemic, the landscape of teaching methodologies continues to be dynamic ^6^. Online education has increased the breadth of information distributed, delivery format flexibility, and access to intended audiences ^6^. Websites allow individuals to promote their “brand” as they want it to be seen, sending potential consumers, or HPE applicants, the message(s) they desire.

Although still a relatively new and expanding field, website reviews of higher education institutions are recognized as an effective way to learn about a program’s identity, culture, values, administrative and organizational structure, available technologies, among others ^7, 8^. In the HPE program space, this theoretically allows HPE programs to appeal to more individuals, and with a more focused and intentional message than before. Given the growing importance of website branding in higher education, it is likely that more time and intention will be placed on the final drafts of HPE programs’ websites ^9^.

Additionally, the growth in the number and diversity of HPE programs is noteworthy. The number of Masters of HPE programs, or an equivalent degree, was cited at 121 in 2014, a staggering growth from a total of 7 in 1999 ^10, 11^. Therefore, Master degrees have become increasingly important to serve as a reference standard to ensure quality educators despite vastly different locations, styles, and content being trained ^5^.

Pugsley *et al.* (2008) tried to gain an understanding of the intra-program differences and found that it varies greatly ^12^. In their 2012 review article, Tekian and Harris noticed the significant growth of masters in HPE programs, and asked some of the same questions we did here ^13^. They explored several concepts, including: geographic location, mission and purpose, program requirements ( *e.g.*, admission requirements, coursework, among others), and program details ( *e.g.*, endpoint recognition, title, tuition costs, duration, instructional strategies, competencies among others). Further, Tekian and Harris’ work called on the HPE community to take action in two areas:

1) Answer an urgent need for expert medical education globally by ensuring that degree-granting programs are geographically distributed to afford individuals with the corresponding competence in HPE
2) Establish criteria for evaluation, including best practices to ensure minimum acceptable quality.

But much has changed since Tekian and Harris’ 2012 study; the world is increasingly technology-driven and competent, the number of HPE programs continues to grow, and HPE as a whole is being given higher recognition for the value it provides the medical field. We used Tekian and Harris’ topics as sensitizing concepts to extract data from websites and chose to continue the discussion of Master-level HPE programs with a different methodology and in greater depth. Thus, our study is bringing to light a novel perspective of HPE masters programs and conveying the voices of the institutions through their websites’ priorities.

Whether as an applicant, an educator, or a researcher, a reasonable starting point to learn what these programs value and teach is via their websites. Websites allow programs to reflect on their priorities and convey them in a manner that they believe adequately represents their program as a whole. This type of widely available advertising gives a unique voice to each institution. We therefore gained insight to these voices through exploration of websites of HPE programs globally and examined their geographical distribution, missions, educational priorities/concentrations, and programmatic components. 

Overall, the purpose of this paper is twofold: First, to demonstrate that websites can be utilized by HPE programs as a way to create a brand and subsequently distribute it to others. Second, this paper demonstrates the use of websites to review the growth and diversity of HPE programs.

## Methods

Our team deduced that most HPE programs would have a website, and that this would serve as a representation of how individuals within the program choose to view themselves and hope to be viewed by others. Further, that these websites would be an efficient means of collecting programmatic information for the purposes of learning more about program growth, diversity, and values. We conducted the website review from August 2021 to April 2022 using a list of worldwide HPE programs, which was acquired from the Foundation of Advancement of International Medical Education and Research’s (FAIMER’s) website ^14^. FAIMER was chosen as the source of programs studied due to its use in another published study evaluating HPE programs ^10^. Each Master’s degree in HPE offered by a university was counted separately, allowing us to note the differences in course and time requirements across all programs. Only HPE masters programs were selected for this study. Certificate and PhD programs were excluded.

Next, we compiled raw data from program websites. All data was collected manually based on what our team thought would be useful for better understanding these institutions. Categories such as programs’ geographical distributions, missions, educational concentrations, and various programmatic logistical components were jointly identified for data collection by three of our authors (JS, SW and HM). Categorization of raw data, into themes and sub-themes, for example, was performed by the same three authors that performed the review, via a joint decision-making process. JS, SW, and HW worked independently through a set of three HPE programs, obtaining the data for these selected categories. Afterward, we manually cross-checked each other's work for verification purposes. For example, if JS obtained the information, SW or HM, who were blinded to JS’s findings, would independently find the answers to the same questions/topics. This was performed until agreement between pre- and post-review information was above 95%. There was no discovered information that was not agreed upon after discussion. Once 100% agreement was reached with this method, the total number of HPE programs analyzed were split between JS and SW and the raw data was obtained for the same categories.

This data then underwent a review by the other two researchers to ensure high accuracy. This review consisted of information verification on individual program websites where it was originally obtained. For example, if JS found the information about a program, SW and HM (now not blinded) would both have to independently find the same information. Any identified discrepancies were rectified through discussion and three-way agreement was mandatory for the team to move on to the next program.

## Results

**Figure 1.**
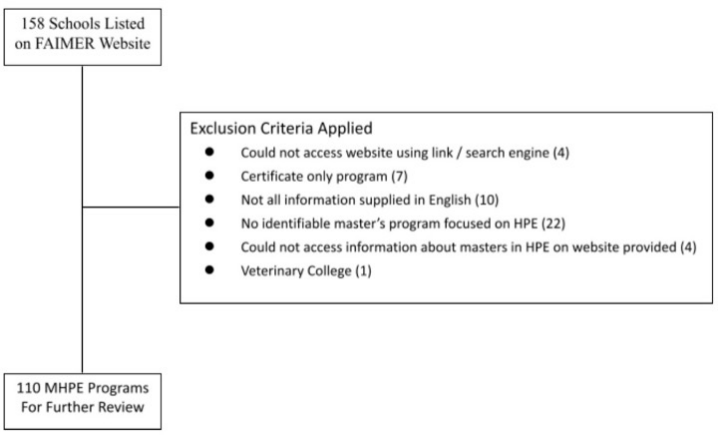
Review Criteria Utilized in MHPE Analysis. This figure is a PRISMA chart used to represent the inclusion and exclusion criteria for review in our Analysis.

After applying the exclusion criteria listed in the PRISMA below ( Figure 1) we were left with 110 masters programs for detailed analysis.

### Geographic location

**Figure 2.**
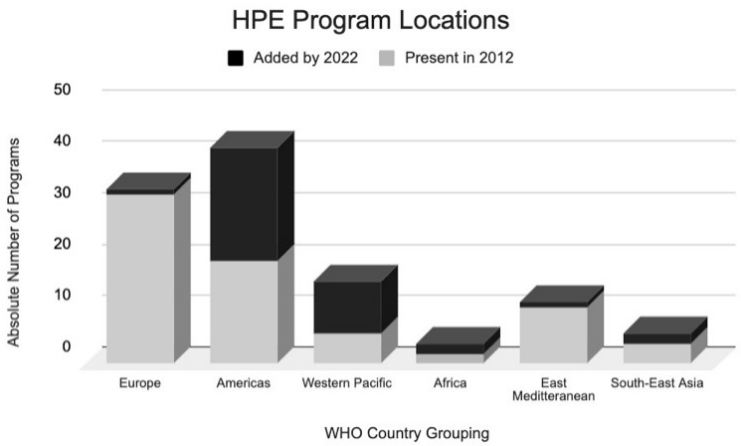
Distribution of HPE programs in absolute numbers in 2012 (Grey) and 2022 (Black). This demonstrates the distribution of all master of Health Profession Educaation programs across the world that were part of our inclusion criteria. The Grey represents programs that were present in 2012 as per Tekian and Harris 2012 review study ^13^. The black blocks represent the additional programs that were added from 2012 to 2022. The categorizations of geographic location are categorized based on WHO member states country groupings for more objective organization, based on a precedent set in the same Tekian and Harris 2012 review ^13^.

Master’s programs were categorized based on WHO member-states country groupings for more objective organization, based on a precedent set by other scholars such as Tekian and Harris in 2012 ^13^. The groupings are in Table III and the distribution of these schools is displayed in Figure 2.

The greatest relative increase was seen in the number of “Western Pacific” programs, which have more than doubled ( Figure 2). A detailed description of the countries included in each WHO classification can be found in Table I, or on the WHO website ^15^. Although in 2012 European programs took up the largest percentage of HPE Master’s programs globally, this rank now belongs to “American” programs ( Figure 2). It is important to notice, that although both “Africa” and “South-East Asia” have doubled their number of programs, they still represent a marginal percentage of these Master’s programs globally. Other interesting trends can be highlighted, such as the significant growth in “American” programs in contrast to the decrease in “African” programs.

**Table I.** WHO classifications as they pertain to countries identified in our HPE research ^15^.

WHO Country Classification	List of Countries
Europe	Austria, Germany, Ireland, Italy, UK, Switzerland, Turkey, Netherlands
Americas	USA, Canada, Cuba, Latin America
Western Pacific	China, Malaysia, Philippians, New Zealand, Australia
Africa	Kenya, South Africa
Eastern Mediterranean	Iran, Pakistan, Saudi Arabia, UAE, Egypt, Sudan
South-East Asia	Indonesia, Bangladesh, India, Thailand

### Mission and purpose

The mission and/or purpose statements of health professions-related Master’s programs vary widely. After solely reviewing the mission or purpose statements from each program, we noted some common themes: education, personal/professional development, research, community improvement, and organizational level skills. Examining each of these themes helped highlight the nuance and variety of the programs. Within the education theme, there was further variation in the form of sub-themes such as: educational theory, teaching practice, teaching and learning skills, assessment, evaluation, design, and curriculum implementation. Within the personal/ professional development theme, we noted topics such as: communication skills, professional development, critical analysis, achieving career goals, well-being of healthcare providers, and reflective practice. The research theme tended to be more unified, and we noted mention of only production of scholarship and performing research. In the community improvement theme, there was mention of: community engagement, diversity, service, clinical care to resource-limited environments, and inclusion. Finally, with regard to the theme of organizational level skills, we noted a propensity of these programs to discuss: preparing leaders, managing change, quality improvement, interprofessional relationships, and mentoring.

### Admissions requirements

Across the 110 programs we reviewed, many different admissions requirements were appreciated. For example, 10 programs (9.09 %) required a professional exam for entrance, while 16 (14.5 %) required previous teaching experience. Complete results of all admission requirements extracted are included in Table III. Notably, only 26 programs (23.6 %) explicitly stated that they require a terminal degree for their applicants. 

### Specialization/concentration

Only a few specializations or concentrations were noted in Tekian and Harris’s 2012 review including: leadership development, clinical education, surgical education, medical education, and HPE more generally ^13^. Our analysis found the field has broadened over the past 10 years with all of the aforementioned specializations/concentrations appearing within our review, as well as some new ones: simulation, philosophy, statistics, ethics, healthcare principles, assessment methods, technology in education, mentoring, research methods, organizational structure and change, academic and professional writing, diversity, quality improvement, and communication.

### Program requirements and duration

Program requirements and their durations varied across the HPE programs analyzed. The average minimum number of years for a master of HPE degree was 2.15 ± 0.88 years for a full-time student. The average advertised range of years typically needed as a part-time student was 4.18 ± 1.45 years. A total of 20 schools (18%) explicitly stated that they offer part-time options within their range of years to completion, while 84 (76%) mentioned it somewhere across their website. We also noted 14 programs (12.7 %) implemented a maximum time frame allowed to complete the masters. Some of these data appear unchanged from previous analysis in 2012, where the average time to completion was “approximately two years” for a full-time student ^13^. The same specific study noted that a part-time student often completed these degrees in a 2–5 year range ^13^.

### Instructional strategies and format

In 2012, Tekian and Harris found that “several programs” offer online and face-to-face education, or a combination of the two ^13^. By 2022, 28 programs (25.5 %) offered their education only in the online format. A total of 13 programs (11.8 %) allowed participants to choose either online or face-to-face format, while 50 programs (45.5 %) were “blended”, allowing a mix of both educational methodologies. Finally, 12 (10.9 **%**) are only face-to-face, while eight programs (7.27 %) published no information on this matter.

Interestingly, it was noted in Tekian and Harris’s 2012 review that one program used simulation along with virtual learning. Although we cannot explicitly state the distribution, it is highly likely that there were more schools that do this in 2022, given the number of programs that teach a course in simulation has grown to 18 (16.4 %).

## Discussion

HPE is an evolving field; the number of programs, their goals, their teaching methodologies, the technology utilized, and many other aspects, remain dynamic. With the increase in graduate HPE programs, it has become important to scan the breadth and focus of these programs and to recognize the impact of programs’ online presence for current and prospective learners and HPE scholars. This study reviewed the websites of all FAIMER graduate HPE Master’s degree programs for two purposes: First, to summarize the current state of HPE programs with regard to geographic distribution, goals and mission, curriculum development and overall programmatic priorities. Second, to complete the review of HPE graduate programs through a website review which affords HPE institutions to represent themselves through the information publicly available on their websites.

Through this study, we found a few pertinent insights that highlight the landscape of HPE Master’s programs as seen through their websites. First, the content HPE programs publish on their websites says a lot about how they view their program, what they offer, what their goals are, and how they intend to progress in the future. Second, the distribution of HPE masters programs is unequally distributed globally. Third, despite being awarded degrees considered to be equitable, these programs are diverging with little to no oversight. Finally, prior to the SARS-CoV-2 pandemic, and accentuated by it, Master’s of HPE institutions are moving towards various teaching modalities, including online learning as a primary means of instruction.

Websites enable programs and prospective students to find each other more easily and ensures alignment of their respective goals and missions. Despite a lack of specific regulatory oversight (there is no accreditation body that specifically accredits HPE programs), by beginning an investigation into these program characteristics, we are starting an important conversation for the development of this field ^5^. Healthcare and education disparities continue to spread across the world, and will continue to worsen as technology plays a more important role in healthcare ^16, 17^. Despite growth in all WHO regions, the relative increase of HPE programs is much more significant in “Europe” and the “Americas” when compared to “South-East Asia”, “Africa”, or the “Western Pacific” ( Table II). This is worth noting because education is correlated with Sustainable Development Goals (SDGs) such as improved gender equality and maternal, newborn, and child health; it is critical that HPE programs do not comply with this geographic maldistribution of education and urgently address this need ^16^.

**Table II.** Data pertaining to distributions of HPE Master’s programs globally ^15^.

	2012 (#,%)	2022 (#, %)
**Europe**	33, 43.42	34, 30.9
**Americas**	20, 26.32	42, 38.2
**Western Pacific**	6, 7.89	16, 14.5
**Africa**	2, 2.63	4, 5.26
**East Mediterranean**	11, 14.5	12, 10.9, 2.63
**South-East Asia**	4, 5.26	2

**Table III.** Raw Data Pertaining to Admissions Requirements of 110 MHPE Programs.

Program Requirements	Number of Programs (#, %)
Required Professional Exam	10, 9.09
Option for non-terminal degree, if with relevant teaching experience	34, 30.9
Terminal Degree	26, 23.6
Relevant Professional Experience	18, 16.4
Teaching experience	16, 14.5
HPE or related degree	32, 29.1
Undergraduate degree (non-related field)	27, 24.5
Dean’s/ Admissions committee exception	4, 3.64
Geographic Limitations	3, 2.73
Statement of Intent	24, 21.8
Active employment (healthcare related) or enrollment in particular job	30, 27.3
No Information Found	5, 4.55

With the number of programs growing, their missions diverging, and minimal oversight across programs, there is a risk of significant educational differences received at one institution versus another, despite being awarded similar degrees. The overview of programs’ specializations/ concentrations suggests that, unsurprisingly, when the missions and/or purposes vary from another program, it is likely that they have differing focus areas. Although we have continued to develop this discussion, a detailed analysis of HPE programs’ coursework and goals/competencies is needed to garner a comprehensive understanding of this issue.

Setting clear expectations helps learners and programs thrive. Graduate HPE websites need to improve the clarity around applicant requirements and, secondarily, come to a consensus on program names and course titles. Do programs named differently truly tend to have differing missions and goals? Do those with the most popular name of “Master of HPE” have similar goals? Our research suggests the former may be true and the latter may be false, however more in-depth analysis is needed to gain a deeper understanding of this claim.

Our research also highlights the importance of transparency in the process of applicant requirements. Given HPE’s status as a Master-level degree, one might reasonably assume that all applicants have a terminal degree of some sort by the time they apply to HPE programs. However, this requirement was not explicitly stated on a majority of websites; unfortunately, this either suggests a lack of transparency or lax academic standards for entry. Furthermore, there was great variation in Master’s graduate HPE program names (see *Underlying data*).

One area where programs were similar throughout our review was their duration for both part and full-time students. Program requirements, on the other hand, were not as streamlined. This, in and of itself, is not necessarily problematic. Whether or not a program decides to use a thesis or final project to culminate their learning experience, or has students take differing combinations of mandatory or elective courses, theoretically does not matter. However, in light of not organizing accreditation structure or standardization to ensure adequate HPE learning, it could be problematic as these programs, and future ones, continue to diverge over time.

It has been generally appreciated that the modem by which education is delivered is gradually becoming more virtual. This was undoubtedly accelerated by the SARS-CoV-2 pandemic ^17^. However, as mentioned in the results, with only 28 MHPE programs (25%) currently offering their degrees online, as well as 50 being blended (45%), it is possible that there is room for future growth into the online domain in this field. One area of particularly interest for research would be to gain a deeper understanding of the effectiveness of MHPE education online versus traditional in-person methods. Assuredly, more research is needed on this topic.

There were several limitations of our study. Although quite extensive, the FAIMER list is not comprehensive. Therefore, there are possibly other programs that would have been identified with a different search strategy. Another limitation is that some programs do not publish their data. We made notes of this above, but the quantity of these absences of data could greatly alter the landscape of what conclusions are reached from a website review of MHPE programs.

Further, information, and often the naming of this information, was often not uniform across HPE programs’ websites, as mentioned above. Therefore, while grouping data based on their similar contexts and inferred meanings, a lack of explicitly coordinated names leaves room for error of interpretation when we re-categorized them under common headings.

As HPE programs continue to grow around the world, in quantity and stature, programs’ ability to advertise effectively online will prove critical to attracting applicants and to sharing their mission and purpose with the broader HPE community. The information published on Master of HPE programs’ websites provides much fruitful data, but there is information missing from these spaces.

## Data Availability

DRYAD: Master in Health Profession Education Website Data, https://doi.org/10.5061/dryad.0zpc86725 ^18^ This project contains the following underlying data: List of MHPE Programs Data for Publication.csv; A list of masters of Health Professions Education programs, or similar, as found online per the above methods section. MHPE Courses Publication Data.csv; Data used to analyze the courses offered t masters of Health Professions Education or similar degrees, as per their online websites. MHPE Missions Publication Data.csv; Data used to analyze the mission statements of masters of Health Professions Education or similar degrees, as per their online websites. MHPE_Program_List_from_FAIMER.csv MHPE_Program_Names.csv MHPE Time Requires Data for Publication.csv; Data used to analyze the time commitments required for masters of Health Professions Education or similar degrees, as per their online websites. Data are available under the terms of the Creative Commons Zero "No rights reserved" data waiver (CC0 1.0 Public domain dedication).
