# Supplementary material for: Masters in health professions education programs as they choose to represent themselves: A website review
Source: MedEdPublish (2016). 2023 Nov 30;13:13. Originally published 2023 Mar 3. [Version 2] doi: 10.12688/mep.19498.2 (PMC10714103; doi:10.12688/mep.19498.2)
Supplement: Peer review history

# Reviewer report: Reviewer response for version 2

**DOI:** 10.21956/mep.21474.r35454

**Published:** 2023-12-11

**Authors:** Michael Wolcott

**Affiliations:** 1 High Point University Workman School of Dental Medicine, North Carolina, USA

No additional comments for the review, thank you!

Have any limitations of the research been acknowledged?

Partly

Is the work clearly and accurately presented and does it cite the current literature?

Yes

If applicable, is the statistical analysis and its interpretation appropriate?

Not applicable

Are all the source data underlying the results available to ensure full reproducibility?

Partly

Is the study design appropriate and is the work technically sound?

Yes

Are the conclusions drawn adequately supported by the results?

Yes

Are sufficient details of methods and analysis provided to allow replication by others?

Partly

Reviewer Expertise:

Program evaluation, curriculum design and development, creative problem solving, assessment

I confirm that I have read this submission and believe that I have an appropriate level of expertise to confirm that it is of an acceptable scientific standard.

# Reviewer report: Reviewer response for version 1

**DOI:** 10.21956/mep.20891.r33989

**Published:** 2023-07-17

**Authors:** Yasser El-Wazir

**Affiliations:** 1 Suez Canal University, Ismailia, Ismailia Governorate, Egypt

Building on a previous similar report published in 2012, this manuscript analyzed the websites of 110 HPE programs based on pre-set criteria of geographic location, mission, admission criteria, and other program characteristics. In general, this provides a useful update of the expansion of HPE programs in different parts of the world and how their online interface can present their identity and some of their educational details.

 I have the following comments to be considered by the authors:

1. I understand that the analysis of mission and purpose was done by thematic analysis; however, authors selected to report the themes that are “common” without identifying how much they are common relative to each other. I think that revealing the frequency of themes can provide more accurate and objective reporting.
2. Data of geographical distribution is unnecessarily repeated in both figure 2 and table 2. I see that the figure is quite illustrative. Table 2 may be deleted or used to summarize data of another studied item.
3. The authors could also comment on the structure of the programs, in terms of number of courses/modules, requirement of a classical thesis, publication or applied project. I think that potential applicants would be interested to know that.
4. I also see that such a manuscript is supposed to be more inclusive, and specifically the language should not be an exclusion criterion because web-based translation tools are common, free and easy to use.
5. I think that the statement in page 4 “the significant growth in “American” programs in contrast to the decrease in “African” programs”, is partially incorrect because the number of African programs actually increased from 2 to 4 as stated in table 2 and figure 2.
6. The manuscript is supposed to end with a meaningful conclusion that conveys useful messages to the HPE programs and their potential applicants.
7. The list of underlying data at the end of the manuscript (page 11) should be hyperlinked to the related webpages. In the same page, there is a typing mistake in the line before the last one.

Have any limitations of the research been acknowledged?

Yes

Is the work clearly and accurately presented and does it cite the current literature?

Yes

If applicable, is the statistical analysis and its interpretation appropriate?

Not applicable

Are all the source data underlying the results available to ensure full reproducibility?

No

Is the study design appropriate and is the work technically sound?

Yes

Are the conclusions drawn adequately supported by the results?

Partly

Are sufficient details of methods and analysis provided to allow replication by others?

Yes

Reviewer Expertise:

Curriculum development, Quality assurance of higher education

I confirm that I have read this submission and believe that I have an appropriate level of expertise to confirm that it is of an acceptable scientific standard, however I have significant reservations, as outlined above.

# Reviewer report: Reviewer response for version 1

**DOI:** 10.21956/mep.20891.r33985

**Published:** 2023-07-17

**Authors:** Michael Wolcott

**Affiliations:** 1 High Point University Workman School of Dental Medicine, North Carolina, USA

Thank you for the opportunity to review. Below are suggestions for revisions.

 INTRODUCTION

- Unsure the first paragraph is necessary, seems very broad and disconnected from the article as a whole.
- I am unsure the purpose of the research is clear. From what it seems, the paper is really a characterization/description of common themes among MHPE programs based on a review of their website. That does not necessarily demonstrate that websites create a brand--that work would require  some additional exploration of brand perceptions by those visiting the sites, analytics, etc. In addition, there is an assumption that information on the website is accurate.

METHODS

- I personally wanted a more exhaustive list of the protocol for collecting data from each website, rather than having to go to the raw data. Unsure if that can be adequately addressed in the limited space, though.
- Can you describe why you excluded certificate and PhD programs? The title may need revision to specifically state this was MHPE programs rather than "graduate health professions education" programs, since that would also include PhDs.
- Why is a veterinary college excludable? Are only "valid" or "quality" MHPE programs offered by schools of medicine? Seems like that limits the scope and potential and also prohibits IPE. Comment about that should be included.
- To clarify, all programs had information about ALL of these components (mission/vision, specialization/concentration, program requirements/duration, and instructional strategies) correct? Otherwise if one piece were missing they would have been excluded--such as the 4 listed in the exclusion criteria. According to the discussion it seems there were absences of data--can you provide an overall estimate of how much data was missing.

DISCUSSION

- Provides a reasonable connection to broader impact, but does not connect back to the original purpose well. I think the purpose itself needs to be re-stated for alignment.
- A significant limitation that does not appear to be stated is an assumption that the information is accurate for the website. It is very easy for programs to offer features that may no longer be present or may not be consistent with the experiences of those participants. An additional research opportunity may be the alignment with actual participant experiences.

Have any limitations of the research been acknowledged?

Partly

Is the work clearly and accurately presented and does it cite the current literature?

Yes

If applicable, is the statistical analysis and its interpretation appropriate?

Not applicable

Are all the source data underlying the results available to ensure full reproducibility?

Partly

Is the study design appropriate and is the work technically sound?

Yes

Are the conclusions drawn adequately supported by the results?

Yes

Are sufficient details of methods and analysis provided to allow replication by others?

Partly

Reviewer Expertise:

Program evaluation, curriculum design and development, creative problem solving, assessment

I confirm that I have read this submission and believe that I have an appropriate level of expertise to confirm that it is of an acceptable scientific standard, however I have significant reservations, as outlined above.

# Reviewer report: Reviewer response for version 1

**DOI:** 10.21956/mep.20891.r32994

**Published:** 2023-03-10

**Authors:** Olle ten Cate

**Affiliations:** 1 Center for Research and Development of Education, University Medical Center Utrecht, Utrecht, The Netherlands

This paper reports on the evaluation of websites that advertise or more generally inform visitors about MHPE programs, to show informative they are and how they meet some earlier published criteria about such programs. I read this manuscript with interest, but felt it has some limitations, some of which may be correctable

1. I would suggest to speak of MHPE programs throughout the manuscript. There are three reasons. (a) “graduate” is any program after a degree, including postgraduate medical education education and that my confuse readers; specifically those from non-North American countries; (b) in the selection of programs, the authors have explicitly excluded PhD programs in their review; towards the end of the manuscript the MHPE abbreviation suddenly emerges – please use this throughout, from the title; and (c) the most fundamental reason is that HPE as used in the paper is confusing and perpetuates its use in a very specific direction. HPE just refers to educational programs for health professions: medicine, nursing, physical therapy, veterinary medicine, et cetera. So the two openings sentences sound odd. Medical education, exists since mankind. We now use a different and more broad term: health professions education, to include nursing, etc. HPE programs are simply programs for training of physicians and other health professionals. So, I propose to correct the terminology throughout the paper and speak of MHPE. Linguistically the abbreviation suffers from a similar flaw, but that I believe is not confusing and widely used with only one meaning
2. In figure 1 I was surprised to see that veterinary MHPEs were excluded, as veterinary medicine belongs to HPE. This also led me to wonder how many of the 110 MHPE programs were not in medical schools but in other HPE schools. I think there are nursing and veterinary MPHE programs and maybe others; can the authors specify that?
3. I was wondering about the admission criteria and the word terminal degree. In Europe, admission to Master’s degrees require the possession of a Bachelor degree (according to the 1999 Bologna agreement – signed by at least 48 countries). But ‘terminal’ degree in academia suggests that there is not a higher degree. But PhD is higher. Or, sometimes in questionnaires, respondents are asked to list ‘their’ terminal degree, with could then be a bachelor (baccalaureate) degree. So what is terminal?
4. The authors find that programs are diverging with little to no oversight.  What does that mean? There programs have not been accredited by and independent body to support their quality? I would be interested to see how many of the 110 MHPE programs are not accredited. In Europe that should not be possible. I would hope that every website for a MHPE would specify the accreditation (preferably with a recent date of approval). And the statement ‘there is no accreditation body that accredits MHPE program’ does not hold for European Master’s programs.
5. I was puzzled by the sentence “the relative increase of HPE programs is much more significant in “Europe” and the “Americas” when compared to “South-East Asia”, “Africa”, or the “Western Pacific”". That contradicts both the figure and the table II.
6. Since this was an international study reporting on world regions, I found it a bit unsatisfactory that all non-English language MHPE programs were excluded. To be critical about geographic maldistribution would require that the authors expand their linguistic scope. They may be right, but how many non-English programs did they encounter that were excluded? Is there something about this the authors can say?
7. I hoped to see something about differences in tuition fees. For many potential applicants of such website the first things you probably want to know are on quality (i.e. accreditation) and costs (tuition fee). This paper could have provided something of a range that would have been helpful for potential applicants.
8. There were some typographical issued. ‘Often’ in a sentence in the penultimate paragraph was uses twice; ‘publish’, page 6, column 2, para 2, should be ‘published’

Have any limitations of the research been acknowledged?

Yes

Is the work clearly and accurately presented and does it cite the current literature?

Yes

If applicable, is the statistical analysis and its interpretation appropriate?

Yes

Are all the source data underlying the results available to ensure full reproducibility?

No

Is the study design appropriate and is the work technically sound?

Yes

Are the conclusions drawn adequately supported by the results?

Partly

Are sufficient details of methods and analysis provided to allow replication by others?

Yes

Reviewer Expertise:

Medical education

I confirm that I have read this submission and believe that I have an appropriate level of expertise to confirm that it is of an acceptable scientific standard, however I have significant reservations, as outlined above.
